# Radiotherapy and Immunotherapy at a Crossroads: Mechanistic Foundations, Emerging Evidence, and a New Horizon for Precision Oncology

**DOI:** 10.3390/jpm16050256

**Published:** 2026-05-08

**Authors:** Gianluca Ferini, Stefano Forte

**Affiliations:** 1 REM Radioterapia srl, 95029 Catania, Italy; 2 Department of Medicine and Surgery, University Kore of Enna, 94100 Enna, Italy; 3 Mediterranean Institute of Oncology, 95029 Catania, Italy; stefano.forte@grupposamed.com

The trajectory of modern oncology is increasingly defined by the convergence of two therapeutic paradigms that were once considered only marginally related: radiotherapy and immunotherapy. For decades, radiotherapy (RT) was perceived primarily as a local treatment, its systemic consequences regarded as incidental rather than intentional. Immunotherapy, by contrast, has been heralded as the modality capable of harnessing or reawakening the body’s own defenses, offering the tantalizing possibility of long-term disease control or even eradication. Yet, the clinical reality has proven more nuanced. Despite the remarkable successes of immune checkpoint inhibitors, adoptive cellular immunotherapy, and various emerging forms of immune-based modulation, the proportion of patients achieving durable responses remains limited. The immune system, even when pharmacologically stimulated, is frequently thwarted by the complexity of tumor microenvironments, by heterogeneity within metastatic lesions, and by the emergence of localized immune escape.

This Special Issue was conceived to illuminate how radiotherapy, particularly when thoughtfully integrated into multimodal strategies, can act as a biological amplifier of antitumor immunity. The contributions assembled here span tumor types that differ profoundly in histology, immune landscape, and clinical behavior, yet collectively reveal a consistent and compelling narrative: radiation has the capacity to reshape the tumor microenvironment, restore immune competence, eliminate resistant clones, and ultimately extend or deepen the therapeutic reach of immunotherapy. The present manuscript collection explores this principle across a spectrum of clinical scenarios, from rare tumors such as Merkel cell carcinoma (MCC), mucosal vaginal melanoma, and glioblastoma, to more common malignancies like renal cell carcinoma (RCC) and squamous cell skin cancers. They also examine how radiotherapy’s immunomodulatory effects may potentiate not only checkpoint inhibitors but also more advanced strategies, including CAR-T cells and next-generation spatially fractionated techniques such as lattice therapy.

What emerges, across this heterogeneous landscape, is a unifying mechanistic foundation that positions radiotherapy as an indispensable partner in the future of immuno-oncology. This editorial aims to integrate and interpret these findings within a narrative framework that captures both the complexity and the translational potential of the field, while also addressing recent advances in cancer vaccines, T-cell lymphomas, and nanoparticle-based theranostic approaches.

**Radiotherapy as a Corrective Force in Immunotherapy Resistance**

One of the clearest themes derived from the contributions of this Special Issue is the crucial role of radiotherapy in addressing oligoprogression during immunotherapy. Oligoprogression, defined as the emergence of limited sites of disease growth while systemic therapy continues to control the broader metastatic burden, has become an increasingly recognized phenomenon as immune checkpoint inhibitors (ICIs) and immune checkpoint inhibitor–tyrosine kinase inhibitor (ICI–TKI) combinations gain prominence. Rather than signaling systemic therapeutic failure, oligoprogression frequently represents the emergence of subclones that have acquired or maintained localized resistance mechanisms [1]. In this context, radiotherapy serves as a corrective force that suppresses these foci of resistance, thereby prolonging the systemic benefit of immunotherapy [2].

The experience reported by Ferini et al. in metastatic MCC illustrates this principle with striking clarity. Their retrospective cohort describes patients experiencing oligoprogression while undergoing treatment with avelumab, a PD-L1 inhibitor that has transformed the management of advanced MCC. Radiotherapy was directed to all sites of progressive disease and was delivered in palliative-dose schedules, most commonly 30 Gy in 10 fractions. The results demonstrate a remarkably high objective response rate of 75%, with 100% local control and survival outcomes that surpass historical expectations in this highly aggressive tumor type. Post-radiotherapy progression-free survival reached 60% at both 6 and 12 months, and overall survival exceeded 85% at one year and more than 64% at two years [3]. These numbers underscore not only the radiosensitivity of MCC but also the profound immunologic collaboration between RT and checkpoint inhibition.

The biological underpinnings of this synergy are multifaceted and well established in the literature. In general, radiation induces immunogenic cell death, increases the availability of tumor antigens, enhances major histocompatibility complex expression, and stimulates dendritic cell maturation, thereby reprogramming the irradiated tumor into an active immunological node that promotes systemic antitumor immunity [4,5]. Yet, the study also expands the conceptual framework of oligoprogression itself. MCC frequently presents with subclinical in-transit metastases and extensive, interconnected cutaneous disease targets that extend beyond conventional oligoprogression definitions based solely on lesion volume and count. Notably, despite the irradiation of very large target volumes (one exceeding 3700 cubic centimeters), the investigators achieved excellent tumor control with minimal toxicity, thereby questioning the adequacy of simple numerical and volumetric parameters as determinants of radiotherapy feasibility [3]. These findings reinforce the notion that radiotherapy can meaningfully extend the efficacy of immunotherapy, even when disease extent complicates traditional clinical decision-making.

A complementary narrative emerges in the work of Lo Greco et al., who analyze the outcomes of patients with advanced or metastatic cutaneous squamous cell carcinoma (CSCC) treated with cemiplimab in combination with radiotherapy. The results here are compelling as well. Across twenty treated lesions, the investigators report an 85% rate of complete or partial response, with no cases of disease progression during follow-up periods extending beyond twenty months. What makes these findings particularly noteworthy is their consistency across different radiation regimens: hypofractionated courses were deployed for frail patients or small-volume disease, while conventionally fractionated schedules were used for larger or more anatomically constrained regions. Regardless of fractionation, the integration of radiotherapy and cemiplimab resulted in consistently favorable clinical outcomes [6].

CSCC is characterized by high tumor mutational burden due to cumulative ultraviolet damage. This biological profile renders the disease intrinsically responsive to immune checkpoint blockade [7]; however, even within this favorable immunogenic context, radiotherapy proved capable of further amplifying treatment response. The combination enabled rapid and sustained tumor regression without introducing additional toxicity; only grade I and II adverse events were observed, reinforcing the feasibility and safety of this synergistic approach [6]. These findings suggest that radiotherapy is not merely a fallback strategy for local control but an active immunologic co-factor capable of intensifying systemic treatment effects.

The study by La Vecchia et al. in metastatic RCC further deepens this argument by examining the role of stereotactic body radiation therapy (SBRT) in patients treated with combination ICI–TKI therapy. RCC has traditionally been considered radioresistant; however, the safe delivery of ultra-hypofractionated high-radiation doses made possible by the development of stereotactic techniques has fundamentally altered this perception. In the setting of oligoprogression, SBRT achieved excellent local control (100% at six months and 70% at one year), while enabling a median next-line systemic treatment-free survival of twenty months.

This metric is especially meaningful, as delaying the need for further systemic therapy reduces both cumulative toxicity and financial burden, extending the period during which patients can maintain a stable quality of life. The investigators emphasize that RT enhances tumor immunogenicity by releasing damage-associated molecular patterns and reducing inter-metastatic seeding, thereby reinforcing the systemic activity of ICIs and TKIs. Their findings consolidate the concept that RT, even in anatomically or biologically challenging tumors, can function as a strategic extension of targeted systemic therapies [8].

**Radiotherapy and Immune Modulation in Rare and Treatment-Resistant Tumors**

Beyond scenarios of oligoprogressive disease, this Special Issue also addresses the interaction between radiotherapy and immune modulation in tumors traditionally regarded as refractory to immunotherapy. Glioblastoma and mucosal vaginal melanoma exemplify malignancies characterized by profound immune suppression, marked intratumoral heterogeneity, and historically limited responsiveness to immune-based treatments. Within this challenging landscape, the contributions collected here do not propose universal solutions but instead delineate the biological boundaries and conditional opportunities through which immune engagement may occur.

Within this spectrum, glioblastoma represents a critical biological limit for radiotherapy-mediated immune modulation. The disease is defined by immune exclusion, dominant myeloid suppression, dysfunctional T-cell trafficking, and a stromal architecture that actively impairs effective immune surveillance [9]. The glioblastoma case included in this Special Issue is therefore informative, not because it establishes a reproducible therapeutic strategy, but because it clarifies the constraints under which radiotherapy operates in this setting. The patient experienced an unexpected and durable regression of a non-resected lesion in the context of prolonged intravesical bacillus Calmette–Guérin (BCG) therapy administered for bladder cancer. As emphasized by the authors, the observed clinical course primarily supports a role for BCG-induced systemic immune activation rather than for radiotherapy itself, which was delivered as part of standard chemoradiotherapy. This observation suggests that, in tumors such as glioblastoma, radiotherapy alone may be insufficient to overcome intrinsic immune resistance and that only exceptionally strong, non-tumor-specific immune stimuli may transiently alter the expected disease trajectory. Importantly, this case does not undermine the rationale for combining radiotherapy with immunotherapy; rather, it suggests that in tumors such as glioblastoma, radiotherapy alone may be insufficient and that achieving therapeutic efficacy likely depends on the presence of additional immune-activating factors [10].

A complementary perspective emerges from the review of mucosal vaginal melanoma, a rare and biologically aggressive malignancy with limited therapeutic options. In contrast to cutaneous melanoma, mucosal variants typically exhibit lower tumor mutational burden, reduced immunogenicity, and inferior responsiveness to immune checkpoint inhibitors [11]. Surgical management is frequently constrained by anatomical considerations, and local recurrence rates remain high [12]. In this context, radiotherapy assumes a central role, not only in achieving local control but also in modulating tumor antigenicity and shaping a microenvironment more permissive to immune infiltration. The reviewed evidence suggests that radiotherapy may synergize with anti-PD-1 therapies, expanding the subset of patients who derive benefit from immunotherapy in a disease historically considered poorly immunogenic. Although high-level prospective data remain limited, these observations support continued investigation into how radiotherapy may recalibrate immune visibility in rare tumors with adverse biological features [13].

**Rewriting the Biology of Solid Tumors: Radiotherapy and CAR-T Cell Therapy**

Among the contributions of this Special Issue, the narrative review on CAR-T cells offers one of the most profound mechanistic insights into the future of immuno-oncology. While CAR-T therapy has achieved transformative success in hematologic malignancies, its application in solid tumors has been consistently limited by a dense and immunosuppressive tumor microenvironment, characterized by heterogeneous and unstable antigen expression [14,15]. Radiotherapy presents a unique opportunity to overcome these challenges, and the review articulates multiple layers of synergy that may ultimately enable the extension of CAR-T therapy into solid tumor settings.

Radiotherapy, depending on dose and fractionation, exerts heterogeneous immunomodulatory forces. High-dose radiation induces immunogenic cell death, increases antigen release, and stimulates the maturation of dendritic cells, creating an inflammatory milieu capable of improving CAR-T cell recognition. Low-dose radiation, by contrast, remodels the tumor stroma, normalizes aberrant vasculature, polarizes macrophages toward an antitumor phenotype, and promotes T-cell infiltration. Both radiation modalities converge on the shared goal of overcoming the barriers that prevent CAR-T cells from trafficking into, persisting within, and effectively destroying solid tumor masses.

The review also highlights the problem of intratumoral hypoxia, a major impediment to CAR-T infiltration and persistence. By modulating vascular flow and reducing hypoxic niches, RT may create a more permissive microenvironment for CAR-T activity. Moreover, the development of hypoxia-responsive CAR constructs represents an elegant example of how synthetic biology may align with radiobiology to refine treatment specificity and enhance safety.

Perhaps the most intriguing element of the review is the exploration of LATTICE radiotherapy, an innovative form of spatially fractionated radiation that delivers high-dose “peaks” to metabolically active tumor areas while maintaining low-dose “valleys” in the surrounding tissue [16,17]. This design preserves vascular integrity, enhances immune trafficking, and generates a rich supply of antigens via immunogenic cell death in the peaks. Early evidence suggests that LATTICE RT can significantly amplify not only CAR-T cell activity but also the efficacy of checkpoint inhibitors, potentially inducing robust systemic immune responses that extend beyond the irradiated field. The authors introduce the concept of “radscopal effects,” wherein the combination of high-dose and low-dose irradiation potentiates immune activation in distant tumor sites, adding a new layer of nuance to the classical abscopal phenomenon.

Collectively, these insights position radiotherapy as an architect of immune permissiveness in solid tumors, capable of dismantling physical and biological barriers that have historically thwarted CAR-T approaches. The implications for future clinical research are profound [18].

**Personalizing DNA Cancer Vaccines**

In this evolving landscape of precision immuno-oncology, the review by Wu et al. provides a comprehensive framework for understanding how personalized DNA cancer vaccines are reshaping antigen-specific immune priming. Moving beyond vaccine strategies based on uniform antigen selection, the authors describe a paradigm in which patient-specific neoantigens, derived from somatic mutations, aberrant splicing events, cryptic translation products, or extrachromosomal DNA, become central drivers of therapeutic specificity. Advances in next-generation sequencing, immunopeptidomics, ribosome profiling, and computational prediction of MHC binding and T-cell receptor recognition are progressively transforming neoantigen discovery from a largely theoretical concept into an increasingly clinically actionable pipeline. Within this framework, DNA-based vaccines emerge as a particularly versatile platform. Compared with RNA-based approaches, they offer molecular stability, favorable safety profiles, prolonged intracellular persistence, and manufacturing flexibility, all of which are critical in the personalized setting. Wu et al. illustrate how advances in vector design and delivery strategies are overcoming historical limitations in nuclear delivery and immunogenicity, while emphasizing that personalized DNA vaccines are most effective when deployed as part of rational combination strategies with immune checkpoint inhibitors, cytokine adjuvants, chemotherapy, and radiotherapy. From the perspective of this Special Issue, this contribution addresses a distinct and essential dimension of precision immunotherapy. Radiotherapy, as discussed throughout this editorial, plays a complementary role by supporting antigen release, enhancing MHC expression, remodeling suppressive tumor microenvironments, and facilitating immune cell infiltration. In this context, Wu et al. note that combining radiotherapy with DNA cancer vaccines can promote antigen presentation and create more permissive conditions for vaccine-induced T-cell responses, while personalized neoantigen selection may help counteract immune escape driven by tumor heterogeneity and clonal evolution. In this sense, personalized DNA vaccines and radiotherapy converge on a shared biological objective, broadening and sustaining antitumor immune pressure across spatially and temporally evolving disease.

Clinical evidence from early-phase studies in triple-negative breast cancer [19], hepatocellular carcinoma [20], glioblastoma [21], melanoma [22], and other solid tumors highlights both the promise and the current limitations of personalized DNA cancer vaccines. The induction of neoantigen-specific T-cell responses, signals of recurrence-free survival benefit in the adjuvant setting, and favorable safety profiles suggest particular relevance in early-stage disease or maintenance strategies. At the same time, Wu et al. critically examine biological barriers, such as immune tolerance, MHC downregulation, T-cell exhaustion, and the immunosuppressive tumor microenvironment, that constrain efficacy in advanced disease and motivate rational combination approaches, including integration with radiotherapy and other immune-modulating interventions.

Overall, precision immunotherapy will increasingly depend on strategies that combine molecular personalization with biological contextualization. Personalized DNA cancer vaccines exemplify this shift, particularly when integrated into multimodal approaches capable of reshaping the tumor microenvironment and sustaining patient-specific immune control [23].

**Personalized Immunotherapy for T-Cell Lymphomas: From Immune Escape to Precision Therapeutics**

In the context of precision immuno-oncology, T-cell lymphomas represent a unique and particularly challenging paradigm. Unlike most solid tumors and B-cell malignancies, these diseases arise from cells that are themselves integral components of the immune system. As comprehensively discussed by Casan et al., this biological paradox profoundly reshapes the conceptual and practical foundations of immunotherapy, transforming immune escape from a secondary resistance mechanism into an intrinsic feature of disease biology.

The review delineates how T-cell lymphomas exploit multiple layers of immune dysfunction, including antigenic ambiguity, phenotypic plasticity, and immune mimicry, to evade therapeutic pressure. Malignant T-cells may downregulate or alter target antigen expression, adopt regulatory or exhausted phenotypes, and interfere with normal immune surveillance, thereby limiting the efficacy of immune-based interventions that rely on intact antitumor immunity. Under these conditions, conventional immunotherapeutic strategies designed to amplify immune responses can paradoxically exacerbate immunosuppression or lead to off-target toxicity, underscoring the need for disease-specific therapeutic logic. From this perspective, the concept of personalization extends well beyond molecular profiling alone. As emphasized by the authors, effective immunotherapy for T-cell lymphomas requires an integrated understanding of tumor subtype, cellular origin, differentiation state, and microenvironmental context. The review highlights how distinct entities, such as cutaneous versus peripheral T-cell lymphomas, exhibit divergent patterns of immune interaction and therapeutic vulnerability, precluding uniform treatment approaches. Personalization therefore emerges as an adaptive process, informed by dynamic tumor–immune interactions rather than static biomarkers.

Casan et al. further examine how contemporary therapeutic strategies attempt to navigate this complexity through rational target selection and combination approaches. Rather than providing an exhaustive catalog of agents, the review focuses on underlying principles guiding the use of immune checkpoint modulation, monoclonal antibodies, epigenetic therapies, and emerging cellular strategies. These interventions are discussed, not as isolated solutions, but as context-dependent tools whose efficacy is shaped by immune escape mechanisms, treatment sequencing, and patient-specific disease characteristics.

Clinical evidence summarized in the review reflects both the promise and the limitations of personalized immunotherapy in T-cell lymphomas. While selected approaches have demonstrated meaningful activity in defined patient subsets, durable responses remain inconsistent, and therapeutic resistance frequently emerges. The authors critically address the biological and immunologic constraints that underlie these outcomes, including T-cell exhaustion, immune tolerance, and the profoundly immunosuppressive tumor microenvironment that characterizes advanced disease. These insights reinforce the notion that therapeutic success in T-cell lymphomas depends less on intensifying immune activation and more on precisely modulating immune context.

From the perspective of this Special Issue, this contribution occupies a distinct and essential thematic space. By interrogating immunotherapy in a setting where the immune system itself is the site of malignant transformation, the review underscores the limits of generalized immunotherapeutic paradigms and highlights the necessity of precision strategies grounded in disease biology. In doing so, it complements the broader narrative of this Special Issue by illustrating that durable immunologic control cannot be achieved through amplification alone, but requires a nuanced understanding of immune escape, adaptability, and context-specific intervention [24].

**Radiolabeled Vitamins and Nanosystems as Potential Agents in Oncology Theranostics**

As precision oncology continues to diversify beyond immunomodulatory approaches, increasing attention has been directed toward theranostic strategies that couple molecular imaging with targeted radionuclide therapy. In this context, the review by Basirinia et al. addresses the development of radiolabeled vitamins and nanosystems as biologically inspired platforms for oncology theranostics, emphasizing their ability to integrate diagnosis, treatment, and response monitoring within a single molecular construct, and to inform treatment personalization in immunologically heterogeneous tumors. The authors focus on vitamins such as folate (vitamin B9) and cobalamin (vitamin B12) as targeting vectors, exploiting physiological uptake mechanisms that are frequently upregulated in cancer cells and within components of the tumor microenvironment. By exploiting receptor-mediated internalization, radiolabeled vitamins offer a rational strategy for tumor selectivity, combining favorable safety profiles with broad applicability across tumor types. Importantly, the review highlights how these targeting strategies may complement immunotherapeutic approaches by refining tumor localization, improving patient stratification, and enabling image-guided combination regimens. Preclinical evidence supporting the use of vitamin-based conjugates paired with diagnostic and therapeutic radionuclides is discussed, illustrating coherent theranostic workflows that can support treatment planning and response assessment. Nanosystems are presented as key technological enablers that extend this concept. Nanoparticles and related carriers provide structural versatility, improved pharmacokinetics, and opportunities for multivalent targeting, including the co-delivery of radionuclides and immunomodulatory agents. Functionalization with vitamins supports active tumor uptake while allowing the incorporation of radionuclides or additional payloads, offering modular solutions to challenges such as tumor heterogeneity, suboptimal biodistribution, and immune-excluded disease niches. Importantly, the review adopts a measured and critical translational perspective. Despite encouraging in vitro and in vivo data, most vitamin- and nanoparticle-based radioconjugates remain in the preclinical domain. Persistent challenges related to radiochemical stability, dosimetry, renal accumulation, and scalable manufacturing are clearly identified as limiting factors for clinical implementation [25,26]. Rather than overstating near-term applicability, the authors delineate realistic development pathways for radiotheranostic platforms, including their potential role within multimodal strategies that integrate targeted radiation delivery with systemic immunotherapy. From the perspective of this Special Issue, this contribution highlights a complementary dimension of precision oncology that intersects with immunotherapy at the level of tumor targeting, microenvironmental modulation, and image-guided treatment adaptation. Radiolabeled vitamins and nanosystems exemplify how advances in nuclear medicine and nanotechnology may converge with immuno-oncology to support patient-specific treatment planning, adaptive therapeutic strategies, and real-time assessment of therapeutic efficacy, reinforcing the notion that precision oncology increasingly relies on the integration of biological insight with technological innovation [27].

**A Convergent Vision for the Future of Immuno-Radiotherapy**

Taken together, the contributions in this Special Issue articulate a coherent vision of radiotherapy not as an ancillary component of cancer treatment but as a central pillar of modern immuno-oncology. Across highly diverse tumors, patient populations, and therapeutic strategies, radiotherapy consistently demonstrates its capacity to deepen and prolong immune responses. In MCC, CSCC, and RCC, RT suppresses escape lesions and extends the lifespan of systemic therapies. In glioblastoma and mucosal vaginal melanoma, it reconfigures profoundly hostile microenvironments, enabling immune interventions that would otherwise be futile. In the realm of adoptive immunotherapy, RT becomes a biological enabler, shaping the conditions in which CAR-T cells might finally realize their potential in solid tumors.

The future of oncologic care will increasingly depend on such integrated strategies. As immunotherapy evolves into ever more personalized and complex modalities, ranging from vaccines and cellular therapies to nanoparticle-guided theranostics, radiotherapy stands as the versatile partner capable of shaping the biological terrain in ways no drug alone can achieve. This Special Issue captures the early contours of this transformation, pointing toward a future in which the boundaries between local and systemic therapy are replaced by a unified therapeutic philosophy grounded in biology, precision, and synergy.
